# The Impact of Disease and Drugs on Hip Fracture Risk

**DOI:** 10.1007/s00223-016-0194-7

**Published:** 2016-09-26

**Authors:** Breiffni Leavy, Karl Michaëlsson, Anna Cristina Åberg, Håkan Melhus, Liisa Byberg

**Affiliations:** 1 Division of Physiotherapy, Department of Neurobiology, Care Sciences and Society, Karolinska Institute, Huddinge, Sweden; 2 Stockholms Sjukhem Foundation, Stockholm, Sweden; 3 Department of Surgical Sciences, Orthopedics, Uppsala University, Uppsala, Sweden; 4 Department of Public Health and Caring Sciences, Geriatrics, Uppsala University, Uppsala, Sweden; 5 School of Education, Health and Social Studies, Dalarna University, Falun, Sweden; 6 Department of Medical Sciences, Osteoporosis and Clinical Pharmacogenetics, Uppsala University, Uppsala, Sweden

**Keywords:** Disease, Dispensed drugs, Hip fracture, Population-based cohort, Risk estimates

## Abstract

We report the risks of a comprehensive range of disease and drug categories on hip fracture occurrence using a strict population-based cohort design. Participants included the source population of a Swedish county, aged ≥50 years (*n* = 117,494) including all incident hip fractures during 1 year (*n* = 477). The outcome was hospitalization for hip fracture (ICD-10 codes S72.0–S72.2) during 1 year (2009–2010). Exposures included: prevalence of (1) inpatient diseases [International Classification of Diseases (ICD) codes A00–T98 in the National Patient Register 1987–2010] and (2) prescribed drugs dispensed in 2010 or the year prior to fracture. We present age- and sex-standardized risk ratios (RRs), risk differences (RDs) and population attributable risks (PARs) of disease and drug categories in relation to hip fracture risk. All disease categories were associated with increased risk of hip fracture. Largest risk ratios and differences were for mental and behavioral disorders, diseases of the blood and previous fracture (RRs between 2.44 and 3.00; RDs (per 1000 person-years) between 5.0 and 6.9). For specific drugs, strongest associations were seen for antiparkinson (RR 2.32 [95 % CI 1.48–1.65]; RD 5.2 [1.1–9.4]) and antidepressive drugs (RR 1.90 [1.55–2.32]; RD 3.1 [2.0–4.3]). Being prescribed ≥10 drugs during 1 year incurred an increased risk of hip fracture, whereas prescription of cardiovascular drugs or ≤5 drugs did not appear to increase risk. Diseases inferring the greatest PARs included: cardiovascular diseases PAR 22 % (95 % CI 14–29) and previous injuries (PAR 21 % [95 % CI 16–25]; for specific drugs, antidepressants posed the greatest risk (PAR 16 % [95 % CI 12.0–19.3]).

**Electronic supplementary material:**

The online version of this article (doi:10.1007/s00223-016-0194-7) contains supplementary material, which is available to authorized users.

## Introduction

Hip fracture is a major cause of disability, dependency and excess mortality among older populations [1, 2], and identification of those at highest risk is of increasing relevance in light of the aging population structure. The vast majority of hip fractures are caused by falls among older people [3, 4], and those with a high prevalence of coexisting disease are more predisposed to falls and hip fracture [5–7]. Hip fracture incidence rises 44-fold in women between age 55 and 85, and the impact by increasing age is 11-fold greater than that associated with the age-related decline in BMD [3]. Preexisting disease, increasing in prevalence with increasing age, may increase hip fracture risk by predisposing to falls or reducing bone strength, or both. In addition, many medications are associated with increased risk of falls and fractures [8, 9], either due to the intended drug effects, or due to unwanted side effects which commonly occur in elderly populations [10]. Diseases affecting the central nervous system (Parkinson’s disease [11], stroke [12], dementia and depression [13, 14], musculoskeletal conditions [15], cardiovascular conditions [12] and bone mineral density [16]) are all implicated in falls and hip fracture occurrence. Drugs affecting the central nervous system, such as sedatives, antidepressants and anticonvulsants, are thought to predispose to falls by slowing cognitive or reflex reaction alertness [8, 17]. These drugs, along with certain cardiovascular drugs, are commonly categorized as fall-risk-increasing drugs [18]. Cardiovascular medications are commonly associated with increased risk of falls and hip fracture due to their propensity to cause or worsen orthostatic hypotension in older people, although evidence for the strength of these associations is differing [9, 19, 20].

There is a scarcity of population-based studies reporting the relative or absolute risks of disease and drugs on hip fracture, and such knowledge would provide important information on the public health impact of diseases and their pharmaceutical treatments on hip fracture occurrence [14, 21]. Cohort studies with recruitment based on clinical examination will fail to identify important disease risk factors due to non-inclusion of the frailest elderly. In addition, self-reported diseases at baseline will be underestimated compared with complete register information [12]. Additionally, by focusing on younger [6] or community-dwelling populations only [22], previous studies have not accounted for the oldest old with highest disease burden. This will in turn produce risk estimates not applicable to residential care dwellers, a group who are at highest risk of falls and account for one-third of all patients with hip fracture [23]. The aim of this study is therefore to report the risks posed by disease and dispensed drugs identified from register data for the incidence of hip fracture, using a strict population-based cohort design.

## Methods

### Subjects and Outcome

All men and women aged 50 years and older (born 1960 and earlier) residing in Uppsala County, Sweden, in 2010 were considered as source population (*n* = 117,494). All incident and radiographically confirmed hip fractures [S72.0–S72.2 according to the International Classification of Diseases 10th revision (ICD-10)] occurring in Uppsala County and admitted to Uppsala University Hospital between June 8, 2009, and June 8, 2010 (*n* = 477), were identified and individually validated by cross-checking of medical records [23]. Pathological (*n* = 5), periprosthetic (*n* = 12) and those fractures where surgical treatment did not occur at Uppsala University Hospital (*n* = 15) were excluded.

The study was approved by the Regional Ethical Review Board in Uppsala. Data concerning the structure of the source population were retrieved from Statistics Sweden’s database (online), available at: www.statistikdatabasen.scb.se/pxweb/sv/ssd/, accessed October 9, 2015.

### Exposures

The National Board of Health and Welfare provided anonymized information on diseases and drugs in sex and age-specific strata (age categories: 50–54, 55–59, 60–64, 65–69, 70–74, 75–79, 80–84, 85+) both for the source population and for those with hip fracture.

### Diseases

Information on diseases was collected from the national patient registry for in-patient care, a nationwide register of all in-patient care in Sweden since 1987. The National Board of Health and Welfare does not hold the register of the total population. The number of persons with record of diseases in the source population was therefore approximated by all records of in-patient care occurring in Uppsala County during the period January 1, 1987–January 1, 2010, among those persons alive on January 1, 2010. For subjects with a hip fracture outcome, information was collected between January 1, 1987, and the day before hip fracture. Information on diagnoses according to ICD-10 chapters was collected: infectious and parasitic diseases (A00–B99); malignant tumors (C00–C97); non-malignant tumors (D00–D48); diseases of blood/blood-forming organs (D50–D89); endocrine, nutritional and metabolic disorders (E00–E90); mental and behavioral disorders (F00–F99); diseases of the nervous system (G00–G99); eye (H00–H59); ear (H60–H95); circulatory system (I00–I99); respiratory system (J00–J99); digestive system (K00–K93); skin and subcutaneous tissue (L00–L99); musculoskeletal system (M00–M99); genitourinary system (N00–N99); previous injury (S00–T98); and fracture injury (S12/S22/S32/42/52/62/72/82), which was a subcategory of injury. Data were extracted such that each diagnosis could be counted once per individual. For subjects with two hip fractures during the observation period (*n* = 7), the date of the second fracture was used as the index date, to prevent diseases being diagnosed more than once for the same individual.

### Drugs

The number of persons being prescribed certain drugs was extracted from the Swedish prescribed drug register. For the source population, this was approximated by all dispensed prescribed drugs registered in Uppsala County during 2010. For subjects with incident hip fracture, all drugs dispensed during 1 year before date of hip fracture were counted. We considered the following drugs or combination of drugs and their ATC (anatomic therapeutic chemical classification system) codes: drugs used in diabetes (A10), systemic corticosteroids (H02), antiparkinson drugs (N04), antidepressives (N06A), benzodiazepines (N05BA); opioid, anxiolytic, hypnotics and sedatives drugs (‘sedatives’; one or more of N02A, N05B-C); cardiovascular system drugs (C); nervous system drugs (N); and fall-risk-increasing drugs defined according to the Swedish National Board of Health and Welfare guidelines [18] which included both psychotropic and cardiovascular drugs (≥1 of N02A, N03A, N04A-B, N05A-C, N06A and ≥1 of C01A, C01BA, C01D, C02, C03, C07–C09, G04CA). The total number of drugs dispensed during 1 year was also assessed and presented as ≥5 drugs/year and ≥10 drugs/year.

### Statistical Analysis

A cohort analysis was performed using the source population as the underlying subjects at risk with the 477 incident hip fractures as outcome. Prevalence of each disease and drug category was calculated stratified by hip fracture outcome. Using risk among those unexposed for each disease or drug category as reference, we calculated risk ratios (RR) and risk differences (RD; risk in the exposed minus the risk in the unexposed, per 1000 person-years) with corresponding 95 % confidence intervals stratified by age (in 5-year strata) and sex and directly standardized to the age and sex distribution of the total population in Uppsala County in 2010 using the ‘cs’ command in Stata. Calculations were additionally done stratified by sex and by age category (50–69; 70–79; 80+ years). The population attributable risk (PAR) (given as a % with corresponding 95 % confidence intervals) was calculated as: prevalence of the exposure among cases multiplied by (1-1/RR) [24]. Statistical analyses were performed using Stata 13.1 (Stata Corp., Collage Station, TX, USA).

## Results

Demographic characteristics of the study population by hip fracture are outlined in Table 1. Women and people 80 years and older were overrepresented among those with hip fracture. Among those aged ≥65 years with hip fracture, 32 % were living in residential care compared to approximately 5 % [25] of those of similar age living in residential care in the region. The 1-year absolute risk of hip fracture in the population aged ≥50 years was 4.1/1000 person-years.

**Table 1:** Characteristics of the study population (*n* = 117 494)

Characteristic, *n* (%)	No hip fracture, *n* = 117 017	Hip fracture, *n* = 477
*Sex*		
Men	56 084 (47.9)	144 (30.2)
Women	60 933 (52.1)	333 (69.8)
*Age, years (%)*		
50–69	80 727 (69.0)	64 (13.4)
70–79	21 502 (18.4)	115 (24.1)
≥80	14,788 (12.6)	298 (62.4)
*Residence* ^a^		
Community (living alone), *n* (%)	Na	144 (30.3)
Community (living with other), *n* (%)	Na	183 (38.4)
Residential care facility, *n* (%)	Na^b^	149 (31.3)

^a^Missing information in one person with hip fracture

^b^Figures available for population in Uppsala County aged ≥65 years 2010: 2628 (4.7 %) [25]

Prevalence of each disease and drug category by hip fracture status and by age group is outlined in Table 2. The most prevalent disease category included circulatory system diseases and previous injury; 59 % of those with hip fracture and 25 % of the remaining population had prevalent circulatory system disease. Corresponding prevalence for previous injury was 43 and 17 %. Drugs in the cardiovascular and nervous system categories were most commonly prescribed. Of note is that 56 % of hip fracture cases had been prescribed sedatives and 52 % prescribed fall-risk-increasing drugs. Prevalence of both diseases and prescribed drugs increased with age.

**Table 2:** Prevalence of diseases and prescribed drugs

	Total population		≤69 years		70–79 years		≥80 years	
	No hip fracture	Hip fracture	No hip fracture	Hip fracture	No hip fracture	Hip fracture	No hip fracture	Hip fracture
	No. (%)	No. (%)	No. (%)	No. (%)	No. (%)	No. (%)	No. (%)	No. (%)
*ICD*-*10 diagnosis chapter*								
Certain infectious and parasitic diseases (A–B)	8052 (6.9)	87 (18.2)	3798 (4.7)	9 (14.1)	1764 (8.2)	12 (10.4)	2490 (16.8)	66 (22.1)
Malignant neoplasms (C)	8452 (7.2)	66 (13.8)	3845 (4.8)	11 (17.2)	2451 (11.4)	19 (16.5)	2156 (14.6)	36 (12.1)
Non-malignant neoplasms (D00-D48)	5674 (4.8)	43 (9.0)	3481 (4.3)	7 (10.9)	1129 (5.3)	11 (9.6)	1064 (7.2)	25 (8.4)
Diseases of the blood/blood-forming organs (D50–89)	4265 (3.6)	80 (16.8)	1631 (2.0)	10 (15.6)	896 (4.2)	15 (13.0)	1738 (11.8)	55 (18.5)
Endocrine, nutritional and metabolic disorders (E)	14,602 (12.5)	148 (31.0)	6839 (8.5)	16 (25.0)	3886 (18.1)	39 (33.9)	3877 (26.2)	93 (31.2)
Mental and behavioral disorders (F)	7971 (6.8)	118 (24.7)	4812 (6.0)	15 (23.4)	1283 (6.0)	26 (22.6)	1876 (12.7)	77 (25.8)
Diseases of the nervous system (G)	7250 (6.2)	90 (18.9)	3532 (4.4)	13 (20.3)	1767 (8.2)	21 (18.3)	1951 (13.2)	56 (18.8)
Diseases of the eye and adnexa (H00-H59)	3883 (3.3)	52 (10.9)	1523 (1.9)	3 (4.7)	944 (4.4)	11 (9.6)	1416 (9.6)	38 (12.8)
Diseases of the ear and mastoid process (H60-H95)	2754 (2.4)	23 (4.8)	1441 (1.8)	2 (3.1)	611 (2.8)	5 (4.3)	702 (4.7)	16 (5.4)
Diseases of the circulatory system (I)	29,018 (24.8)	283 (59.3)	12,690 (15.7)	20 (31.3)	7730 (36.0)	66 (57.4)	8598 (58.1)	197 (66.1)
Diseases of the respiratory system (J)	10,682 (9.1)	117 (24.5)	5198 (6.4)	14 (21.9)	2485 (11.6)	19 (16.5)	2999 (20.3)	84 (28.2)
Diseases of the digestive system (K)	18,450 (15.8)	144 (30.2)	10,726 (13.3)	12 (18.8)	3860 (18.0)	31 (27.0)	3864 (26.1)	101 (33.9)
Diseases of the skin and subcutaneous tissue (L)	3543 (3.0)	23 (4.8)	1977 (2.4)	3 (4.7)	727 (3.4)	8 (7.0)	839 (5.7)	12 (4.0)
Diseases of the musculoskeletal system and connective tissue (M)	17,953 (15.3)	151 (31.7)	9181 (11.4)	12 (18.8)	4234 (19.7)	32 (27.8)	4538 (30.7)	107 (35.9)
Diseases of the genitourinary system (N)	15,853 (13.5)	154 (32.3)	8037 (10.0)	12 (18.8)	3415 (15.9)	32 (27.8)	4401 (29.8)	110 (36.9)
Injury, poisoning and certain other consequences of external causes (S–T)	19,399 (16.6)	203 (42.6)	10,559 (13.1)	25 (39.1)	4010 (18.6)	42 (36.5)	4830 (32.7)	136 (45.6)
Fracture of the neck, thorax, spine, arm, hip, leg, ankle (S12/S22/S32/42/52/62/72/82)	9722 (8.3)	148 (31.0)	4576 (5.7)	20 (31.3)	2007 (9.3)	30 (26.1)	3139 (21.2)	98 (32.9)
*Drug (ATC code)*								
Drugs used in diabetes (A10)	10,686 (9.1)	57 (11.9)	5714 (7.1)	6 (9.4)	2964 (13.8)	21 (18.3)	2008 (13.6)	30 (10.1)
Corticosteroids systemic (H02)	7984 (6.8)	41 (8.6)	4542 (5.6)	8 (12.5)	1922 (8.9)	13 (11.3)	1520 (10.3)	20 (6.7)
Antiparkinson drugs (N04)	2027 (1.7)	26 (5.5)	879 (1.1)	4 (6.3)	563 (2.6)	11 (9.6)	585 (4.0)	11 (3.7)
Benzodiazepines (N05BA)	9018 (7.7)	103 (21.6)	4178 (5.2)	7 (10.9)	2004 (9.3)	19 (16.5)	2836 (19.2)	77 (25.8)
Opioid, anxiolytic, hypnotics and sedatives drugs^a^	33,119 (28.3)	267 (56.0)	18,048 (22.4)	25 (39.1)	7115 (33.1)	61 (53.0)	7956 (53.8)	181 (60.7)
Antidepressant drugs (N06A)	16,006 (13.7)	162 (34.0)	9332 (11.6)	14 (21.9)	2909 (13.5)	35 (30.4)	3765 (25.5)	113 (37.9)
Cardiovascular system drugs (C)	59,165 (50.6)	362 (75.9)	31,529 (39.1)	35 (54.7)	14,670 (68.2)	87 (75.7)	12,966 (87.7)	240 (80.5)
Nervous system drugs (N)	48,342 (41.3)	356 (74.6)	27,216 (33.7)	38 (59.4)	10,207 (47.5)	80 (69.6)	10,919 (73.8)	238 (79.9)
Fall-risk-increasing drugs^b^	23,990 (20.5)	250 (52.4)	10,338 (12.8)	19 (29.7)	6081 (28.3)	55 (47.8)	7571 (51.2)	176 (59.1)
Five or more dispensed drugs during 1 year^c^	57,005 (48.7)	394 (82.6)	29,877 (37.0)	44 (68.8)	13,933 (64.8)	90 (78.3)	13,195 (89.2)	260 (87.2)
Ten or more dispensed drugs during 1 year^c^	25,030 (21.4)	249 (52.2)	10,698 (13.3)	25 (39.1)	6684 (31.1)	56 (48.7)	7648 (51.7)	168 (56.4)

Prevalence of diseases in the in-patient register between 1987 and 2010 for the total population (approximated by all in-patient care registered in Uppsala County for patients alive January 1, 2010) and between 1987 and day before date of fracture for subjects with hip fracture. Prevalence of prescribed drugs from the prescribed drug register in 2010 for the total population (approximated by all prescribed drugs registered in Uppsala County for patients alive January 1, 2010) and for 1 year before date of fracture for subjects with hip fracture

^a^One or more of N02A, N05B-C

^b^One or more of N02A, N03A, N04A-B, N05A-C, N06A AND one or more of C01A, C01BA, C01D, C02, C03, C07-C09, G04CA

^c^Any ATC code

No major differences were observed across disease exposures between men and women, and a combined estimate is therefore presented although estimates stratified by sex are given in Supplementary Table 1.

**Fig. 1:**
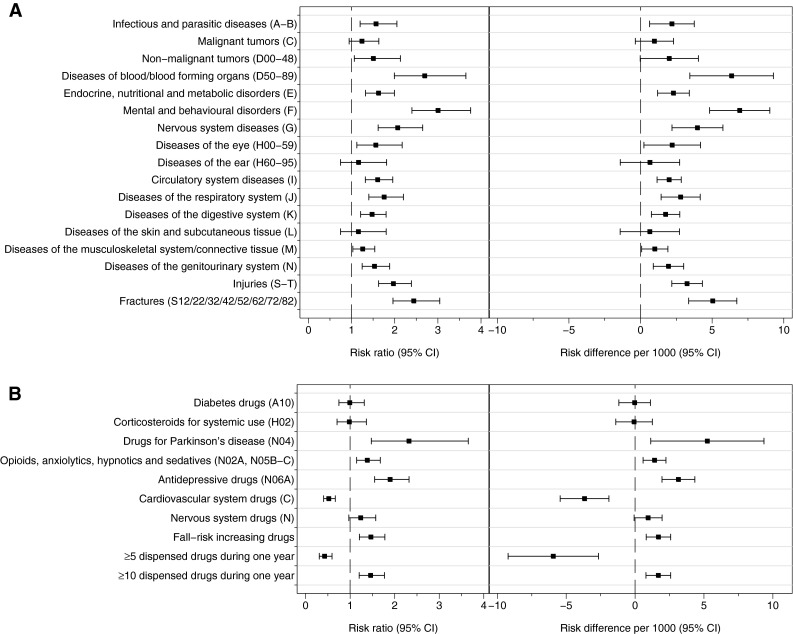
Standardized risk ratios and risk differences (per 1000) and their 95 % confidence intervals for the association of prevalent in-patient treated disease between 1987 and 2010 (**a**) and of dispensed drugs in 2010 (**b**) with incident hip fracture

To estimate the association between each disease category and hip fracture risk, age- and sex-standardized risk ratios (RR) and differences (RD) were calculated (Fig. 1a). All disease categories were associated with an increased risk of hip fracture, and highest RRs were observed for mental and behavioral disorders (RR 3.00, 95 % CI 2.40–3.76), diseases of the blood (RR 2.70, 95 % CI 1.99–3.65) and previous fractures (RR 2.44, 95 % CI 1.96–3.04). An approximately twofold increase in risk was seen for previous injuries and disorders of the nervous system (Fig. 1a). An approximate 1.6-fold increase in risk was observed for more prevalent conditions such as circulatory system disorders (RR 1.61, 95 % CI 1.32–1.95) and endocrine disorders (RR 1.63, 95 % CI 1.33–1.99). Conditions with largest risk differences for hip fracture occurrence (per 1000 person-years) included mental and behavioral disorders (6.9), diseases of the blood (6.4), previous fractures (5.0) and diseases of the nervous system (4.0). In relation to age, RRs for all disease categories were highest among the youngest age groups (50–69 years) and decreased with increasing age although risk differences were lower. For example, having a mental and behavioral disorder was associated with a fivefold increase in risk among those 50–69 years with a risk difference of 3/1000 person-years; for the age group 70–79, the risk was increased fivefold (risk difference 15/1000 person-years), and for the oldest age group (80+), there was a twofold increased risk with a risk difference of 17/1000 person-years (Table 3).

**Table 3:** Standardized risk ratios and risk differences (per 1000) and their 95 % confidence intervals for the association of prevalent in-patient treated disease between 1987 and 2010 and of dispensed drugs in 2010 with incident hip fracture by age

	≤69 years		70–79 years		≥80 years	
	RR	RD/1000	RR	RD/1000	RR	RD/1000
*ICD*-*10 diagnosis chapter*						
Certain infectious and parasitic diseases (A–B)	3.28 (1.60 to 6.71)	1.6 (0.1 to 3.2)	1.36 (0.75 to 2.48)	1.9 (−2.3 to 6.0)	1.30 (0.99 to 1.71)	5.6 (−0.8 to 12.1)
Malignant neoplasms (C)	2.89 (1.48 to 5.66)	1.3 (0.1 to 2.6)	1.50 (0.91 to 2.45)	2.5 (−1.0 to 6.0)	0.85 (0.60 to 1.19)	−3.1 (−9.2 to 2.9)
Non-malignant neoplasms (D00-D48)	3.54 (1.51 to 8.30)	1.9 (−0.3 to 4.0)	1.54 (0.83 to 2.88)	2.8 (−2.0 to 7.6)	1.08 (0.72 to 1.62)	1.6 (−6.9 to 10.0)
Diseases of the blood/blood-forming organs (D50-89)	7.64 (3.83 to 15.24)	4.5 (1.2 to 7.9)	3.27 (1.90 to 5.62)	10.9 (2.9 to 19.0)	1.51 (1.12 to 2.04)	9.5 (1.5 to 17.5)
Endocrine, nutritional and metabolic disorders (E)	2.81 (1.58 to 4.97)	1.2 (0.3 to 2.1)	2.28 (1.55 to 3.36)	5.5 (2.3 to 8.8)	1.19 (0.93 to 1.51)	3.5 (−1.7 to 8.7)
Mental and behavioral disorders (F)	5.13 (2.88 to 9.14)	2.7 (1.0 to 4.3)	4.52 (2.93 to 6.99)	15.5 (7.8 to 23.1)	2.02 (1.55 to 2.63)	17.6 (9.3 to 25.8)
Diseases of the nervous system (G)	4.61 (2.48 to 8.55)	2.4 (0.7 to 4.1)	2.50 (1.55 to 4.01)	7.1 (1.9 to 12.3)	1.43 (1.07 to 1.91)	7.9 (0.6 to 15.2)
Diseases of the eye and adnexa (H00-H59)	1.80 (0.56 to 5.80)	0.6 (−1.0 to 2.2)	2.34 (1.25 to 4.36)	6.7 (−0.3 to 13.8)	1.22 (0.87 to 1.71)	4.2 (−3.6 to 12.0)
Diseases of the ear and mastoid process (H60-H95)	1.44 (0.35 to 5.90)	0.3 (−1.2 to 1.9)	1.42 (0.58 to 3.47)	2.2 (−4.4 to 8.8)	1.01 (0.61 to 1.66)	0.1 (−9.9 to 10.1)
Diseases of the circulatory system (I)	1.85 (1.06 to 3.22)	0.6 (−0.0 to 1.2)	2.47 (1.70 to 3.59)	5.2 (2.8 to 7.6)	1.29 (1.02 to 1.65)	5.0 (0.5 to 9.5)
Diseases of the respiratory system (J)	3.67 (2.02 to 6.66)	1.8 (0.5 to 3.1)	1.47 (0.90 to 2.40)	2.3 (−1.1 to 5.8)	1.49 (1.16 to 1.92)	8.9 (2.6 to 15.3)
Diseases of the digestive system (K)	1.38 (0.74 to 2.60)	0.3 (−0.3 to 0.9)	1.70 (1.12 to 2.56)	3.3 (0.3 to 6.3)	1.41 (1.11 to 1.79)	7.4 (1.9 to 12.8)
Diseases of the skin and subcutaneous tissue (L)	1.93 (0.60 to 6.20)	0.7 (−1.0 to 2.4)	2.02 (0.99 to 4.14)	5.2 (−2.0 to 12.5)	0.69 (0.38 to 1.23)	−6.3 (−14.6 to 1.9)
Diseases of the musculoskeletal system and connective tissue (M)	1.45 (0.77 to 2.71)	0.3 (−0.3 to 1.0)	1.50 (1.00 to 2.26)	2.4 (−0.3 to 5.2)	1.13 (0.89 to 1.43)	2.5 (−2.4 to 7.4)
Diseases of the genitourinary system (N)	1.81 (0.93 to 3.51)	0.6 (−0.2 to 1.4)	2.01 (1.33 to 3.02)	4.6 (1.3 to 8.0)	1.30 (1.03 to 1.64)	5.4 (0.3 to 10.5)
Injury, poisoning and certain other consequences of external causes (S–T)	4.03 (2.44 to 6.65)	1.7 (0.8 to 2.6)	2.41 (1.65 to 3.51)	5.9 (2.7 to 9.0)	1.46 (1.16 to 1.83)	7.8 (2.8 to 12.8)
Fracture of the neck, thorax, spine, arm, hip, leg, ankle (S12/S22/S32/42/52/62/72/82)	6.53 (3.85 to 11.09)	3.2 (1.5 to 4.9)	3.12 (2.04 to 4.76)	9.2 (4.2 to 14.3)	1.49 (1.16 to 1.92)	8.8 (2.7 to 14.9)
*Drug (ATC code)*						
Drugs used in diabetes (A10)	1.28 (0.55 to 3.01)	0.2 (−0.6 to 1.1)	1.47 (0.91 to 2.36)	2.3 (−1.0 to 5.7)	0.76 (0.53 to 1.11)	−4.8 (−10.8 to 1.2)
Corticosteroids systemic (H02)	2.19 (1.03 to 4.66)	0.9 (−0.3 to 2.1)	1.30 (0.73 to 2.31)	1.5 (−2.3 to 5.3)	0.63 (0.40 to 1.00)	−7.5 (−13.6 to −1.4)
Antiparkinson drugs (N04)	5.80 (2.01 to 16.79)	3.6 (−0.9 to 8.1)	4.29 (2.30 to 7.98)	16.3 (3.7 to 28.8)	0.91 (0.50 to 1.65)	−1.8 (−12.6 to 8.9)
Benzodiazepines (N05BA)	1.73 (0.78 to 3.85)	0.6 (−0.4 to 1.6)	1.92 (1.15 to 3.22)	4.6 (−0.0 to 9.2)	1.22 (0.94 to 1.59)	4.2 (−1.6 to 10.0)
Opioid, anxiolytic, hypnotics and sedatives drugs^a^	1.96 (1.18 to 3.26)	0.6 (0.1 to 1.2)	2.13 (1.47 to 3.08)	4.3 (2.0 to 6.7)	1.07 (0.85 to 1.36)	1.5 (−3.2 to 6.2)
Antidepressant drugs (N06A)	1.96 (1.06 to 3.63)	0.7 (−0.1 to 1.5)	2.76 (1.84 to 4.14)	7.6 (3.4 to 11.8)	1.58 (1.25 to 1.99)	10.0 (4.3 to 15.7)
Cardiovascular system drugs (C)	1.34 (0.81 to 2.21)	0.2 (−0.2 to 0.6)	1.38 (0.90 to 2.12)	1.6 (−0.4 to 3.6)	0.36 (0.27 to 0.48)	−32.1 (−45.4 to −18.8)
Nervous system drugs (N)	2.63 (1.59 to 4.36)	0.8 (0.4 to 1.3)	2.30 (1.54 to 3.44)	4.3 (2.3 to 6.4)	0.86 (0.64 to 1.16)	−3.4 (−10.2 to 3.5)
Fall-risk-increasing drugs^b^	2.09 (1.22 to 3.59)	0.7 (0.1 to 1.4)	2.23 (1.54 to 3.22)	4.9 (2.3 to 7.5)	1.12 (0.89 to 1.41)	2.3 (−2.3 to 7.0)
Five or more dispensed drugs during 1 year^c^	2.83 (1.65 to 4.84)	0.8 (0.4 to 1.2)	1.75 (1.12 to 2.74)	2.7 (0.8 to 4.7)	0.26 (0.18 to 0.37)	−54.4 (−79.7 to −29.1)
Ten or more dispensed drugs during 1 year^c^	3.09 (1.85 to 5.16)	1.2 (0.5 to 2.0)	2.01 (1.39 to 2.90)	4.1 (1.7 to 6.5)	1.04 (0.83 to 1.30)	0.7 (−3.8 to 5.3)

^a^One or more of N02A, N05B-C

^b^One or more of N02A, N03A, N04A-B, N05A-C, N06A AND one or more of C01A, C01BA, C01D, C02, C03, C07–C09, G04CA

^c^Any ATC code

For drugs dispensed over 1 year (Fig. 1b), the largest RRs and RDs (per 1000 person-years) were observed for antiparkinson drugs (RR 2.32, 95 % CI 1.48–3.65; RD 5.2, 95 % CI 1.1–9.4) and antidepressive drugs (RR 1.90, 95 % CI 1.55–2.32; RD 3.1, 95 % CI 2.0–4.3), whereas diabetes drugs incurred no increased risk (RR 0.99, 95 % CI 0.75–1.32). Benzodiazepines (RR 1.46, 95 % CI 1.14–1.87) and other sedative drugs (RR 1.39, 95 % CI 1.15–1.68) were also associated with an increased risk of hip fracture. Being prescribed fall-risk-increasing drugs (a combination of cardiovascular and nervous system drugs) was associated with 47 % increased risk of hip fracture (RR 1.47, 95 % CI 1.21–1.78). However, the overall estimate for cardiovascular drugs on hip fracture was 0.52 (95 % CI 0.41–0.67). The age-stratified analysis revealed that this is driven by the RR in the highest age group with RRs of 1.34 (0.81–2.21) and 1.38 (0.90–2.12) for age groups ≤69 and 70–79 years, respectively (Table 3). A similar pattern was seen for being prescribed 5 or more drugs during 1 year; the RR for hip fracture was 2.83 (1.65–4.84) for those aged ≤69 years (Fig. 1b; Table 3). Being prescribed 10 or more drugs during 1 year was associated with an increased risk of hip fracture (Fig. 1b, Table 3). The age-related decline in relative risk for drugs was not as established a pattern as that for diseases, although RRs were lower in general in the oldest age category (≥80 years) than in the youngest (≤69 years).

In order to establish the proportion in the overall population attributable to disease or drug exposures, we calculated the PAR. For disease exposures, highest PARs occurred for cardiovascular diseases (22.4 %, 95 % CI 14.4–29.0), injuries (21.0 %, 95 % CI 16.4–24.7), previous fracture (18.3 %, 95 % CI 15.2–20.8) and mental and behavioral disorders (16.5 %, 95 % CI 14.4–18.2). PAR for diseases of the blood was 10.6 % (95 % CI 8.4–12.2). Specific drugs incurring the highest attributable risks at the population level were antidepressives (PAR 16.1 %, 95 % CI 12.1–19.3) and sedative drugs (PAR 15.6 %, 95 % CI 7.1–22.6), in addition to fall-risk-increasing drugs (PAR 16.7 %, 95 % CI 9.0–23.0) and more than 10 drugs dispensed during 1 year (PAR 16.4 %, 95 % CI 8.8–22.7). Given a low prevalence, PAR for antiparkinson drugs was only 3.1 % (95 % CI 1.8–4.0).

## Discussion

We assessed the relative and absolute risk of hip fracture during 1 year based on the entire population of a Swedish County in relation to a wide range of disease and drug categories. Hospitalization for any disease posed an increased risk of future hip fracture, with largest risk estimates seen for mental and behavioral disorders, diseases of the blood and previous fracture. Prescriptions of antiparkinson or antidepressive drugs were associated with largest increased risk of hip fracture.

The main strengths of the study include the capturing and subsequent verification of all incident hip fractures during 1 year in a Swedish county. The patient register covers all in-patient care in Sweden since 1987, and as reporting is mandatory, disease recording is of high quality [26]. The prescribed drug register includes information on all prescription medicines dispensed at Swedish pharmacies, hospitals and residential care facilities. Both registers allow individual matching using the personal identification number from all Swedish residents. Information on diseases and dispensed drugs was collected for the source population and those with hip fracture during the 1-year study period, allowing for a true population-based cohort analysis. Data were provided by the National Board of Health and Welfare in sex and age strata. However, since the National Board of Health and Welfare does not hold the population register, exposures in the source population were approximated by counting all in-patient treated diseases in Uppsala County occurring between 1987 and 2010 among those still alive January 1, 2010, and by counting all drugs dispensed in Uppsala County during 2010 in an open cohort design. This provides a fair approximation of the total population if it is assumed that inflow and outflow of inhabitants are equal. However, it also is a potential source for overestimation of the prevalence of disease in the source population, especially in the oldest ages, which would lead to a conservative bias of our estimates. The disease exposures will not account for all diagnoses made in outpatient settings which may lead to a conservative bias toward the null of the prevalence of conditions commonly treated in out-patient settings, such as diabetes, dementia, depression or hypertension [26]. This would nonetheless have been the case for both hip fracture patients and the total population. In contrast, osteoporosis would have been more commonly diagnosed in the in-patient register only among those with history of a previous fall-related fracture. Using age- and sex-standardized risk ratios for exposures on a population level, we were not able to estimate the effects independent of potential covariates such as body mass index, bone mineral density, smoking or demographic factors. Neither could we examine the effects of disease onset or severity or length of treatment with certain drugs. Furthermore, we cannot tell whether individuals had a combination of diseases or, in the case of fall-related drugs and 5 or 10 drugs prescribed during 1 year, whether the prescribed drugs were taken simultaneously or not. Additionally, a dispensed drug does not necessarily mean that the drug was taken.

We observed positive associations between all disease categories and hip fracture, a pattern reported by others, albeit for a smaller range of diseases, in relation to falls [11] and hip fracture [21]. The threefold increased risk of hip fracture associated with mental and behavioral disorders, the most prevalent of which are disorders caused by psychoactive substances, mood disorders, schizophrenia and dementia, is in accordance with the twofold to threefold increased risk observed in previous cohort studies [14, 21]. Dementia and hip fracture share several predisposing and intermediate risk factors, such as advanced age, gait impairments and increased risk of falls [27]. We also observed an increased risk of hip fracture for hypnotics and sedative drugs, commonly prescribed to people with dementia, which are previously reported to adversely affect postural stability and judgment and further predispose to injurious falls [17], particularly during nighttime hours [23]. Our findings involving a doubled risk of hip fracture associated with antidepressant drugs correspond to results from previous studies [17, 28]. Others report that antidepressants are the most frequently dispensed fall-related drugs among elderly people in primary care and in institutional settings [29]. Additionally, the population attributable risk of 16 % that these medications infer is an important reminder of the need for restrictive prescription of antidepressants among elderly people at risk of falls.

Antiparkinson drugs incurred the greatest relative and absolute risks of hip fracture of all drugs investigated, despite a low prevalence in the population, as also indicated by the population attributable risk of 3 %. Dopaminergic medications effectively treat motor impairments and gait performance in Parkinson’s disease [30]. They are, however, also reported to negatively affect postural adjustments [31] and sway, both factors that could predispose to fall-related hip fractures. We cannot separate from our results the effects of disease from drug treatment of disease, and the observed reductions in RR estimates for antiparkinson drugs with increasing age (Table 3) may therefore reflect either reduced exposure to falls due to reduced mobility, or reduced effectiveness of dopaminergic agents, both factors that occur in the advanced disease stages [32].

Previous fracture is a known risk factor for future hip fracture [33]. We report a RR of 2.44 (95 % CI 1.96–3.04) for previous fractures which is slightly higher than that reported in a meta-analysis of 11 cohort studies (RR 1.86, 95 % CI 1.75–1.98) [34]. Our method of registering only previous fractures which required hospitalization leads to a channeling toward more severe fractures, which more strongly predict future hip fracture than minor fractures cared for in out-patient settings or captured through self-reported methods. In consideration that the vast majority of injury-related hospital admissions among elderly people are fall related [3] and those who fall frequently are at greatest risk of facture outcomes, hip fracture prevention may begin by posing simple screening questions relating to whether an older person has fallen in the previous year or has an impaired balance [35].

We observed that diseases of the blood inferred an approximate threefold increased risk of hip fracture, with a population attributable risk of 10 %. A slightly lower risk estimate for the effect of pernicious anemia (vitamin B12 deficiency) on hip fracture has been reported previously [36, 37]. Although vitamin B12 deficiency might negatively influence bone strength [38], it may not be the sole mechanism. Deficiency anemias are common in hip fracture patients [39]. Anemia in general is most prevalent among institutionalized elderly people and in the oldest old, and has been associated with reduced physical performance and falls [40, 41]. Among the current hip fracture cohort, we were able to ascertain that 469 out of 477 hip fractures (98 %) were preceded by a fall. Thus, only in eight patients, specific fall circumstances were not possible to identify [23]. In addition, anemia may increase the severity of other preexisting diseases [40] or influence risk of hip fracture via other, yet unknown mechanisms. Our results highlight the importance of investigating the presence of diseases of the blood, conditions that are often treatable, among at-risk groups as a part of fracture prevention.

Surprisingly, cardiovascular drugs and having been dispensed five drugs or more during 1 year, were associated with lower risk of hip fracture. The finding that cardiovascular drugs were associated with reduced risk of hip fracture appears somewhat contradictory due to their reported hypotensive effects among older people, a symptom that commonly precedes hip fracture in the clinical context [42]. Nonetheless, there are previous studies reporting weak associations between cardiovascular drugs and falls [9] and hip fractures [43, 44]. It is also possible that the apparent protective effect of cardiovascular drugs occurs due to our strict population-based cohort, which includes residential care dwellers with the poorest health, who are reported less likely to receive cardiovascular drugs when compared with community dwellers [45]. These differences in prescription are proposed to be due to a lack of adherence to treatment guidelines, as opposed to lower cardiovascular disease prevalence in this group [46]. Indeed, the inverse association in our study seems to be driven by the inverse association in the oldest age category, whereas in younger ages prescribed cardiovascular drugs are associated with increased risk of hip fracture. Nevertheless, we report that circulatory system diseases were associated with a doubled risk of hip fracture, which falls within the wide range of estimates reported for stroke and heart failure, estimates that vary in relation to age, sex and time since disease onset in previous studies [12, 47]. Furthermore, cardiovascular disease posed the largest population attributable risk (22 %).

Interestingly, we observed that being prescribed five or more medications during 1 year did not infer an increased overall risk of hip fracture, whereas being prescribed ten or more medications during 1 year did. However, as with cardiovascular drugs, this inverse association was driven by the highest age group as an increased risk of hip fracture was seen for those younger than 80 years. One possible interpretation could be that in our cohort, the use of up to five drugs is a marker for health-seeking behavior or for community dwellers with a relatively lower disease burden, since the drug register enabled us to establish the number of dispensed medications during 1 year, but not the extent to which these drugs were taken simultaneously. The concomitant use of more than five drugs, on the other hand, has been shown to be twice as common among institutionalized elderly in Sweden, a group with frailer health status [45]. Additionally, those receiving a greater number of drugs are also more likely to receive psychotropic fall-risk-increasing drugs [48, 49] which could further explain the higher RRs for excessive polypharmacy in our findings. The literature regarding the effects of polypharmacy on hip fracture is conflicting, possibly reflecting the complex underlying mechanisms affecting the relationship between polypharmacy and fracture risk which include: confounding effects of disease, nature of the drugs, and/or interaction effects between specific drugs. A recent nested case–control study reported higher odds ratios for hip fracture when taking two or more drugs [50]. This is in contrast to positive relationships between polypharmacy and injurious falls reported in a cohort study, when only in the presence of established fall-related drugs [51].

In a population-based setting of adults aged 50 and older, the 1-year risk of hip fracture is higher among those that previously were hospitalized for any disease but especially so for mental and behavioral disorders, diseases of the blood and previous injury. Additionally, being prescribed antiparkinson, antidepressive, sedative or ten or more medications during 1 year, all associate with increased risk of hip fracture. Using a strict population-based cohort, we were able to report the impact of a wide range of disease and drug categories on risk of hip fracture. Our results add to the few existing studies which have included the frailest members of the elderly population when calculating the impact of disease and drugs on hip fracture risk.

## Electronic supplementary material

Below is the link to the electronic supplementary material.

Supplementary material 1 (DOCX 16 kb)
