# Supplementary material for: The Impact of Disease and Drugs on Hip Fracture Risk
Source: Calcif Tissue Int. 2016 Sep 26;100(1):1–12. doi: 10.1007/s00223-016-0194-7 (PMC5214955; doi:10.1007/s00223-016-0194-7)
Supplement: Supplementary file 1 — Supplementary material 1 (DOCX 16 kb) [file 223_2016_194_MOESM1_ESM.docx]

SUPPLEMENTAL MATERIAL

Supplementary Table 1. Prevalence, Standardized Risk Ratios and Risk Differences (per 1000) and Their 95% Confidence Intervals for the Association of Prevalent in-Patient Treated Disease between 1987 and 2010 (panel A) and of Dispensed Drugs in 2010 (panel B) with Incident Hip Fracture by Sex

|  | **Women** | | | | **Men** | | | |
| --- | --- | --- | --- | --- | --- | --- | --- | --- |
|  | **No hip fracture** | **Hip fracture** |  |  | **No hip fracture** | **Hip fracture** |  |  |
|  | **No. (%)** | **No. (%)** | **RR** | **RD/1000** | **No. (%)** | **No. (%)** | **RR** | **RD/1000** |
| ***ICD-10 diagnosis chapter*** |  |  |  |  |  |  |  |  |
| Certain infectious and parasitic diseases (A-B) | 4099 (6.7) | 63 (18.9) | 1.67 (1.22 to 2.30) | 3.5 (0.8 to 6.1) | 3953 (7.0) | 24 (16.7) | 1.33 (0.80 to 2.19) | 0.8 (-0.8 to 2.4) |
| Malignant neoplasms (C) | 4846 (8.0) | 46 (13.8) | 1.16 (0.85 to 1.59) | 0.9 (-1.0 to 2.8) | 3606 (6.4) | 20 (13.9) | 1.42 (0.84 to 2.40) | 1.1 (-0.8 to 2.9) |
| Non-malignant neoplasms (D00-D48) | 4315 (7.1) | 34 (10.2) | 1.39 (0.98 to 1.98) | 2.1 (-0.5 to 4.6) | 1359 (2.4) | 9 (6.3) | 1.77 (0.84 to 3.70) | 1.9 (-1.3 to 5.2) |
| Diseasese of the blood/blood forming organs (D50-89) | 2583 (4.2) | 54 (16.2) | 2.34 (1.64 to 3.32) | 6.8 (2.8 to 10.8) | 1682 (3.0) | 26 (18.1) | 3.57 (2.06 to 6.22) | 5.9 (1.6 to 10.1) |
| Endocrine, nutritional and metabolic disorders (E) | 8004 (13.1) | 103 (30.9) | 1.52 (1.19 to 1.95) | 2.6 (0.9 to 4.4) | 6598 (11.8) | 45 (31.3) | 1.88 (1.31 to 2.69) | 1.9 (0.6 to 3.3) |
| Mental and behavioural disorders (F) | 4335 (7.1) | 84 (25.2) | 2.74 (2.09 to 3.59) | 8.1 (5.0 to 11.2) | 3636 (6.5) | 34 (23.6) | 3.63 (2.43 to 5.41) | 5.6 (2.9 to 8.4) |
| Diseases of the nervous system (G) | 3604 (5.9) | 54 (16.2) | 1.79 (1.30 to 2.45) | 4.0 (1.3 to 6.8) | 3646 (6.5) | 36 (25.0) | 2.79 (1.87 to 4.17) | 3.9 (1.7 to 6.1) |
| Diseases of the eye and adnexa (H00-H59) | 2076 (3.4) | 39 (11.7) | 1.56 (1.07 to 2.26) | 2.9 (-0.0 to 5.9) | 1807 (3.2) | 13 (9.0) | 1.57 (0.80 to 3.07) | 1.4 (-1.2 to 4.0) |
| Diseases of the ear and mastoid process (H60-H95) | 1562 (2.6) | 19 (5.7) | 1.46 (0.89 to 2.38) | 2.5 (-1.3 to 6.2) | 1192 (2.1) | 4 (2.8) | 0.49 (0.19 to 1.32) | -1.3 (-2.6 to -0.0) |
| Diseases of the circulatory system (I) | 13812 (22.7) | 192 (57.7) | 1.53 (1.22 to 1.93) | 2.4 (1.1 to 3.8) | 15206 (27.1) | 91 (63.2) | 1.79 (1.23 to 2.60) | 1.5 (0.5 to 2.5) |
| Diseases of the respiratory system (J) | 5509 (9.0) | 78 (23.4) | 1.58 (1.20 to 2.07) | 2.9 (0.9 to 4.9) | 5173 (9.2) | 39 (27.1) | 2.19 (1.47 to 3.27) | 2.7 (0.9 to 4.5) |
| Diseases of the digestive system (K) | 9542 (15.7) | 95 (28.5) | 1.41 (1.11 to 1.79) | 2.0 (0.5 to 3.6) | 8908 (15.9) | 49 (34.0) | 1.64 (1.15 to 2.34) | 1.4 (0.3 to 2.6) |
| Diseases of the skin and subcutaneous tissue (L) | 2004 (3.3) | 17 (5.1) | 1.13 (0.68 to 1.88) | 0.7 (-2.4 to 3.8) | 1539 (2.7) | 6 (4.2) | 1.23 (0.52 to 2.89) | 0.6 (-2.1 to 3.3) |
| Diseases of the musculoskeletal system and connective tissue (M) | 10477 (17.2) | 115 (34.5) | 1.24 (0.98 to 1.56) | 1.2 (-0.2 to 2.6) | 7476 (13.3) | 36 (25.0) | 1.31 (0.89 to 1.93) | 0.8 (-0.4 to 1.9) |
| Diseases of the genitourinary system (N) | 10313 (16.9) | 110 (33.0) | 1.50 (1.19 to 1.89) | 2.4 (0.9 to 4.0) | 5540 (9.9) | 44 (30.6) | 1.61 (1.07 to 2.42) | 1.4 (-0.0 to 2.8) |
| Injury, poisoning and certain other consequences of external causes (S-T) | 10533 (17.3) | 151 (45.3) | 1.94 (1.54 to 2.43) | 4.2 (2.5 to 5.8) | 8866 (15.8) | 52 (36.1) | 2.05 (1.44 to 2.91) | 2.2 (0.9 to 3.6) |
| Fracture of the neck, thorax, spine, arm, hip, leg, ankle (S12/S22/S32/42/52/62/72/82) | 5937 (9.7) | 115 (34.5) | 2.24 (1.74 to 2.89) | 5.8 (3.4 to 8.1) | 3785 (6.7) | 33 (22.9) | 2.91 (1.93 to 4.39) | 4.2 (1.8 to 6.6) |
|  |  |  |  |  |  |  |  |  |
| ***Drug (ATC code)*** |  |  |  |  |  |  |  |  |
| Drugs used in diabetes (A10) | 4550 (7.5) | 42 (12.6) | 1.15 (0.83 to 1.60) | 0.8 (-1.2 to 2.8) | 6136 (10.9) | 15 (10.4) | 0.65 (0.38 to 1.12) | -0.9 (-1.9 to 0.1) |
| Corticosteroids systemic (H02) | 4802 (7.9) | 24 (7.2) | 0.72 (0.48 to 1.10) | -1.5 (-3.3 to 0.2) | 3182 (5.7) | 17 (11.8) | 1.62 (0.96 to 2.73) | 1.5 (-0.5 to 3.6) |
| Antiparkinson drugs (N04) | 1151 (1.9) | 18 (5.4) | 1.86 (1.13 to 3.07) | 4.6 (-0.3 to 9.5) | 876 (1.6) | 8 (5.6) | 3.40 (1.51 to 7.65) | 6.0 (-0.8 to 12.7) |
| Benzodiazepines (N05BA) | 5927 (9.7) | 82 (24.6) | 1.47 (1.12 to 1.93) | 2.4 (0.5 to 4.4) | 3091 (5.5) | 21 (14.6) | 1.43 (0.85 to 2.41) | 1.1 (-0.7 to 2.8) |
| Opioid, anxiolytic, hypnotics and sedatives drugs^a^ | 20593 (33.8) | 200 (60.1) | 1.28 (1.02 to 1.61) | 1.4 (0.1 to 2.7) | 12526 (22.3) | 67 (46.5) | 1.63 (1.16 to 2.30) | 1.4 (0.4 to 2.5) |
| Antidepressant drugs (N06A) | 10725 (17.6) | 118 (35.4) | 1.63 (1.29 to 2.05) | 3.0 (1.4 to 4.6) | 5281 (9.4) | 44 (30.6) | 2.58 (1.78 to 3.76) | 3.3 (1.5 to 5.0) |
| Cardiovascular system drugs (C) | 30827 (50.6) | 251 (75.4) | 0.42 (0.32 to 0.56) | -7.2 (-10.4 to -4.0) | 28338 (50.5) | 111 (77.1) | 1.05 (0.64 to 1.72) | 0.1 (-1.1 to 1.4) |
| Nervous system drugs (N) | 29768 (48.9) | 257 (77.2) | 1.01 (0.76 to 1.35) | 0.1 (-1.6 to 1.8) | 18574 (33.1) | 99 (68.8) | 1.98 (1.35 to 2.90) | 1.9 (0.9 to 2.9) |
| Fall-risk increasing drugs^b^ | 14369 (23.6) | 178 (53.5) | 1.23 (0.98 to 1.55) | 1.2 (-0.1 to 2.5) | 9621 (17.2) | 72 (50.0) | 2.14 (1.52 to 3.03) | 2.2 (1.1 to 3.4) |
| 5 or more dispensed drugs during one year^c^ | 32635 (53.6) | 281 (84.4) | 0.35 (0.24 to 0.52) | -10.5 (-16.5 to -4.6) | 24370 (43.5) | 113 (78.5) | 0.77 (0.44 to 1.33) | -0.9 (-3.1 to 1.2) |
| 10 or more dispensed drugs during one year^c^ | 15125 (24.8) | 182 (54.7) | 1.36 (1.08 to 1.71) | 1.8 (0.4 to 3.2) | 9905 (17.7) | 67 (46.5) | 1.71 (1.20 to 2.45) | 1.6 (0.4 to 2.7) |

a (one or more of N02A, N05B-C)

b (one or more of N02A, N03A, N04A-B, N05A-C, N06A AND one or more of C01A, C01BA, C01D, C02, C03, C07-C09, G04CA)

c (any ATC code)
